# Glycosyltransferase Family 43 Is Also Found in Early Eukaryotes and Has Three Subfamilies in Charophycean Green Algae

**DOI:** 10.1371/journal.pone.0128409

**Published:** 2015-05-29

**Authors:** Rahil Taujale, Yanbin Yin

**Affiliations:** Department of Biological Sciences, Northern Illinois University, DeKalb, Illinois, United States of America; The University of Melbourne, Australia

## Abstract

The glycosyltransferase family 43 (GT43) has been suggested to be involved in the synthesis of xylans in plant cell walls and proteoglycans in animals. Very recently GT43 family was also found in Charophycean green algae (CGA), the closest relatives of extant land plants. Here we present evidence that non-plant and non-animal early eukaryotes such as fungi, *Haptophyceae*, *Choanoflagellida*, *Ichthyosporea* and *Haptophyceae* also have GT43-like genes, which are phylogenetically close to animal GT43 genes. By mining RNA sequencing data (RNA-Seq) of selected plants, we showed that CGA have evolved three major groups of GT43 genes, one orthologous to IRX14 (IRREGULAR XYLEM14), one orthologous to IRX9/IRX9L and the third one ancestral to all land plant GT43 genes. We confirmed that land plant GT43 has two major clades A and B, while in angiosperms, clade A further evolved into three subclades and the expression and motif pattern of A3 (containing IRX9) are fairly different from the other two clades likely due to rapid evolution. Our in-depth sequence analysis contributed to our overall understanding of the early evolution of GT43 family and could serve as an example for the study of other plant cell wall-related enzyme families.

## Introduction

Xylans are the second most abundant polysaccharides in plant cell walls. Much progress has been made in elucidating the biosynthesis of xylans in model plant organisms and has been most recently reviewed by [1]. Briefly using genetic screening mutants with irregular xylem phenotype, two GT43 proteins (AtIRX9 and AtIRX14) and one GT47 protein (AtIRX10) in *Arabidopsis thaliana* were suggested to be involved in the synthesis of the xylan backbone [2–6]. Further studies have shown that their close paralogous proteins (IRX9-like, IRX14-like and IRX10-like) might function redundantly [2, 3]. A recent study hypothesized that IRX9, IRX14 and IRX10 might work together as a large protein complex [1, 7]. Lastly, other GT proteins particularly those of GT47 and GT8 families, were also indicated to play a role in xylan backbone synthesis [2, 8, 9].

In addition to functional characterizations, phylogenetic and sequence motif studies of GT43 proteins have also been published to understand the evolution and origin of the GT43 family. Two reports have included non-plant proteins in their analyses and demonstrated that plant and animal proteins are phylogenetically well separated [10, 11]. One of the reports showed that three major GT43 clades (monophyletic clusters) exist in land plants [10]: A contains AtIRX9L, B contains AtIRX14/14L and C contains AtIRX9. More recent studies in early plants suggested that there are only two major land plant GT43 clades [12, 13], as AtIRX9 and AtIRX9L were grouped into one single clade named A. Early land plants (moss and spike moss) [12] and Charophycean green algae (CGA) [13] were shown to have orthologous genes in each of the two clades. We have previously identified a single chlorophyte green algal GT43 homolog (GenBank ID: XP_003063890.1) from *Micromonas pusilla* CCMP1545 using a hidden Markov model (HMM)-based approach [14], and have demonstrated that clade A could be further split into three subclades, with AtIRX9 and AtIRX9L in two separate subclades and the third subclade containing no Arabidopsis genes.

Clearly there exist some disagreements in the literature with regard to the phylogenetic classification of the GT43 family. One of the possible reasons might be that the analyzed data in previous reports were from different organisms: e.g. some only used land plants, while others also included CGA or even animals. We believe that including proteins of broader organismic groups in the phylogeny could help improve the classification resolution. In addition, although AtIRX14/14L clade (B) and AtIRX9/9L clade (A) must have already separated since CGA [13], it is unknown when AtIRX9 and AtIRX9L separated. CGA transcriptomes have been recently mined for different cell wall-related GT families [13]; however only a very small number of GT43 hits were phylogenetically analyzed due to short sequence lengths of assembled transcripts. Therefore it remains unanswered how many GT43 clades exist in CGA and how are they evolutionarily related to land plant clades. Furthermore, since a distant GT43 homolog was found in chlorophyte green algae [14] that evolved earlier than CGA, it is possible that GT43 genes might be present in other early organisms.

In recent years, the study of the evolution of plant cell walls has gained considerable interest [15–17], as it can assist in understanding the complexity of cell wall compositions across different plant lineages and the information used to develop transgenic plants with improved biomass. Here we sought to further the evolutionary study of the GT43 family among all cellular kingdoms, as it is difficult to believe that GT43’s are absent in more ancient early non-plant/algal/animal eukaryotes and prokaryotes. We aimed to obtain a more robust phylogenetic classification of plant/algal GT43 genes by including more representative species especially those without complete genomes such as liverworts, hornworts, ferns and CGA. We used more sensitive bioinformatics approaches combining sequence homology searches, conserved motif scanning and expression profiles to better understand the evolutionary divergence of the GT43 family.

## Results and Discussion

### GT43-like proteins are found in non-plant/animal early eukaryotes but not in prokaryotes

In order to look for non-*Streptophyta* and non-*Metazoa* GT43 homologs, we have performed an exhaustive HMMER search (www.hmmer.org) against the NCBI non-redundant protein database (NCBI-nr), the largest protein sequence database containing both experimentally and computationally determined proteins. The query is an HMM profile of the GT43 family downloaded from dbCAN database [18]. In total 21 non-*Streptophyta* and non-*Metazoa* protein homologs (Table 1, including the previously reported XP_003063890.1 of chlorophyte *M*. *pusilla* CCMP1545 with E-value = 2.3e-22) were found using a very conservative cutoff E-value < 1e-10. A BLASTP search of these 21 proteins against the CAZy database [19] showed that all candidates had a GT43 protein as their best hit.

**Table 1:** 21 NCBI-nr proteins that are non-plant and non-animal proteins and hit the GT43 domain with an E-value < 1e-10; GenBank identifiers (IDs) having at least 3 motifs are shown in bold and italic.

GenBank ID	Length	Species	Taxonomic group	GT43 domain E-value	GenBank ID of best BLAST hit in CAZy database	E-value of best hit in CAZy database	Total # of motifs *
***XP_004345176*.*1***	**390**	***Capsaspora owczarzaki ATCC 30864***	***Ichthyosporea***	**1.50E-59**	**CAI62038.1**	**4.80E-25**	**6**
***XP_001744836*.*1***	**305**	***Monosiga brevicollis MX1***	***Choanoflagellida***	**3.60E-56**	**AFE77696.1**	**6.80E-42**	**6**
***XP_001742263*.*1***	**243**	***Monosiga brevicollis MX1***	***Choanoflagellida***	**1.80E-48**	**AEO35548.1**	**6.00E-39**	**6**
***XP_001747805*.*1***	**280**	***Monosiga brevicollis MX1***	***Choanoflagellida***	**7.50E-48**	**AAI69407.1**	**4.10E-37**	**6**
***XP_007514541*.*1***	**352**	***Bathycoccus prasinos***	***Chlorophyta***	**9.30E-28**	**CAI62044.1**	**2.00E-09**	**5**
***XP_004343171*.*1***	**458**	***Capsaspora owczarzaki ATCC 30864***	***Ichthyosporea***	**2.30E-51**	**CAI63873.1**	**2.90E-18**	**4**
***XP_004989824*.*1***	**324**	***Salpingoeca rosetta***	***Choanoflagellida***	**1.80E-50**	**AAH78400.1**	**3.00E-31**	**4**
***EMS23236*.*1***	**318**	***Rhodosporidium toruloides NP11***	***Fungi***	**1.90E-33**	**CAA15837.2**	**1.40E-14**	**4**
***EGU10941*.*1***	**318**	***Rhodotorula glutinis ATCC 204091***	***Fungi***	**1.70E-32**	**CAA15837.2**	**8.80E-15**	**4**
***XP_005794278*.*1***	**290**	***Emiliania huxleyi CCMP1516***	***Haptophyceae***	**5.00E-34**	**ACF84760.1**	**2.80E-08**	**3**
***XP_003336254*.*2***	**221**	***Puccinia graminis f sp tritici CRL 75-36-700-3***	***Fungi***	**2.40E-24**	**CAB04033.2**	**1.30E-07**	**3**
XP_003336238.1	472	*Puccinia graminis f sp tritici CRL 75-36-700-3*	*Fungi*	1.80E-23	CAB04033.2	4.40E-08	2
AIA87534.1	116	*uncultured bacterium*	*Bacteria*	1.90E-13	AAI21201.1	4.40E-13	2
XP_004345780.1	370	*Capsaspora owczarzaki ATCC 30864*	*Ichthyosporea*	6.30E-37	AAI21202.1	4.50E-14	1
EFW41058.2	407	*Capsaspora owczarzaki ATCC 30864*	*Ichthyosporea*	8.30E-37	AAI21202.1	8.10E-14	1
XP_005769385.1	547	*Emiliania huxleyi CCMP1516*	*Haptophyceae*	2.80E-31	CAI93173.1	1.90E-08	1
XP_003063890.1	227	*Micromonas pusilla CCMP1545*	*Chlorophyta*	2.30E-22	CAI63866.1	6.40E-05	1
AIA83381.1	112	*uncultured bacterium*	*Bacteria*	3.00E-14	CAI63870.1	3.20E-25	1
XP_003336242.2	158	*Puccinia graminis f sp tritici CRL 75-36-700-3*	*Fungi*	2.80E-11	ABF18200.1	1.80E-03	1
XP_003325987.2	529	*Puccinia graminis f sp tritici CRL 75-36-700-3*	*Fungi*	9.90E-21	CAI68027.1	1.10E-03	0
XP_007416303.1	119	*Melampsora larici-populina 98AG31*	*Fungi*	4.40E-13	CAI68027.1	1.20E-02	0

* Motif details are available in S1 Table

If using a more relaxed cutoff E-value < 1e-5, 428 additional non-*Streptophyta* and non-*Metazoa* hits were found (i.e. with E-value between 1e-5 and 1e-10), 380 of which are from prokaryotes, 27 are from fungi, 14 are from other eukaryotes (*Alveolata*, *Euglenozoa*, *Chlorophyta*) and 8 are from viruses. However a BLASTP search against the CAZy database showed that only four of the 428 proteins had a GT43 protein as their best hit, suggesting that most of them are false positives. Furthermore a manual examination of the four proteins (all from *Trypanosoma cruzi* of *Euglenozoa*) by inspecting NCBI Blink (pre-computed BLAST result against NCBI-nr) pages and conserved domain search pages suggested that none of them seem to be GT43 like.

**Fig 1:**
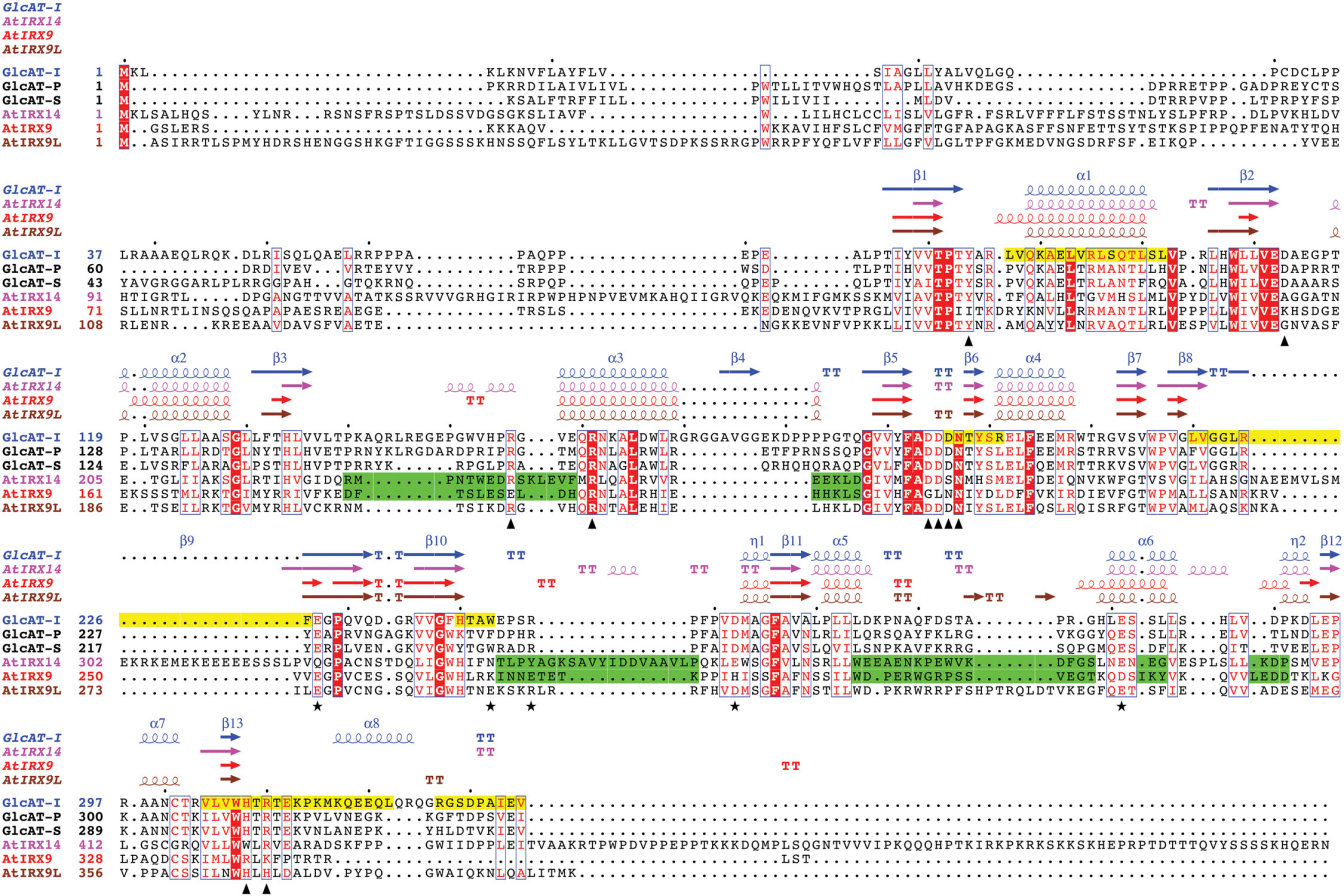
Sequence alignment of human and Arabidopsis GT43 proteins. Human proteins GlcAT-I (UniProt ID: O94766), GlcAT-P (Q9P2W7), GlcAT-S (Q9NPZ5) and Arabidopsis proteins AtIRX9, AtIRX9L and AtIRX14 were used to build the alignment using MAFFT program [41]. The alignment graph was generated using ESPript server [45], which also took the PDB format files of GlcAT-I (PDB ID: 1kws), AtIRX9 (predicted), AtIRX9L (predicted) and AtIRX14 (predicted) to display their secondary structures. Arrows and stars below the alignment indicate residues in GlcAT-I structure that interact with UDP-GlcA (sugar donor) and the trisaccharide Galβ1-3Galβ1-4Xyl (sugar acceptor). Yellow shaded regions indicate protein-protein interaction (PPI) regions reported in GlcAT-I structure [21]. Green shaded regions indicate weakly aligned regions that do not overlap with PPI regions.

To be more conservative, we further scanned the 21 protein sequences (Table 1 and detailed version in S1 Table) for the presence of 8 PROSITE-style [20] motif patterns shown in Table 2. These 8 patterns were derived by referring to a previous report [11] covering 17 key residues (S2 Table and Fig 1) interacting with the substrates of a structurally solved human GT43 protein named GlcAT-I [21]. Because these motif patterns were defined very strictly, our automatic motif scanning approach is very conservative and having these motifs is a strong support for a protein to be a GT43 protein. Finally 11 out of the 21 proteins have at least 3 of the 8 motifs and all of the 11 proteins have GT43 domains with very significant E-values (between 2.4e-24 and 1.5e-59).

**Table 2:** PROSITE [20] style patterns of 8 conserved sequence motifs found in GT43 proteins.

Motif	PROSITE pattern	Position in human GlcAT-I *
1	T-P-[TI]-[YI]	81–84
2	W-[IL]-[ILV]-[VIA]-E-[DAKG]	108–113
3	[QMN]-R-[NL]	160–162
4	D-D-[DS]-N	194–197
5	[EQ]-[GA]-P	227–229
6	[ILVM]-[DEH]-[MWI]-[AS]-[GS]-F	251–256
7	[QLN]-[DE]-[SNT]	280–282
8	W-[HRNW]-[LT]-[RQKH]	307–310

* Details are available in S2 Table and Fig 1

These 11 proteins (bold and italic GenBank IDs in Table 1) belong to eight species of five taxonomic groups: *Choanoflagellida*, *Ichthyosporea*, *Fungi*, *Chlorophyta* and *Haptophyceae*. The other 10 of the 21 proteins (Table 1) have less than 3 motifs but are also from these five taxonomic groups, except for two proteins (AIA87534.1 and AIA83381.1) that are annotated as being derived from uncultured bacteria. Both AIA87534.1 and AIA83381.1 are short in sequence (< 120 aa) and NCBI Blink pages show that the top 100 hits of both proteins are all from *Metazoa*. Given that the two proteins were derived from intestinal metagenomes of snout beetle (*Rhynchophorus ferrugineus*) [22], it is likely that they are actually encoded by beetle genes but were mis-annotated as deriving from uncultured intestinal bacteria. As mentioned above, 380 prokaryotic proteins have E-values between 1e-5 and 1e-10; however Blink and conserved domain examination indicated that their top hits are not GT43 proteins. Hence we can conclude that no prokaryotic GT43 homologs were found in our search and GT43 is a family restricted to eukaryotes.

We believe that the absence of GT43 homologs in prokaryotes can be explained as follows. GT43 might have evolved from other GT families in that many GT families, including GT43, GT2 and GT8, are classified into the GT-A superfamily in the CAZy database [19]. The reason that we do not see homology between eukaryotic GT43 proteins and prokaryotic GT-A proteins is because they have diverged too much in sequence although the structural similarity can still be detected. In fact, of the aforementioned 428 additional protein hits with E-value between 1e-5 and 1e-10, 67 bacterial proteins matched other GT proteins (excluding GT43) as their best hit in the CAZy database, among which 42 had a GT2 as their best hit.

### GT43 proteins are found in CGA, liverworts, hornworts and ferns

Using the same approach, we mined RNA sequencing (RNA-Seq) data in GenBank generated from 5 liverworts, 2 hornworts, 3 ferns and 15 CGA (S3 Table). Although these plants are expected to have GT43 genes, we wanted to prove that GT43 genes are indeed present in these plant taxa and then further study how they are phylogenetically related to flowering plant GT43 genes. We modified a computational pipeline that we developed recently [23] to assemble RNA-Seq reads species by species and then mined these assembled transcripts for GT43 homologs (S1 Fig).

Particularly for CGA, a previous report [13] used pre-assembled expressed sequence tag (EST) data. We on the other hand started with the raw RNA-Seq reads because pre-assembled ESTs usually only cover a subset of the original RNA-Seq reads. In addition, the previous report used ESTs sequenced mostly by Sanger and 454 technologies. We however employed ~166 million reads (S3 Table) sequenced using the Illumina technology, an approach that is known to have increased sequencing depth. These together determined that we would be able to retrieve more GT43 homologs that might have been missed by the previous analysis.

Using an E-value < 1e-10 in a HMMER search, 54 transcript hits were obtained. A BLASTP search of these 54 transcripts against the CAZy database showed that all of them hit a GT43 protein as the best match. After further applying the motif filter, 29 peptide sequences translated from the 54 transcripts (S4 Table), including 6 from liverworts, 3 from hornworts, 3 from ferns and 17 from CGA, have at least 3 of the 8 motifs. A total of 21 have an amino acid sequence length > 180.

### Phylogenetic classification of GT43 family in all organismic groups

In order to understand how the newly identified GT43 homologs are phylogenetically related to land plant and animal proteins, we combined sequences of 12 early eukaryotic proteins (Table 1, 11 proteins with 3+ motifs plus XP_003063890.1 of the chlorophyte green alga), 29 early plant peptides (S4 Table) as well as 33 plant and animal proteins selected from CAZy and PlantCAZyme [24] databases to build a maximum likelihood phylogeny (see details in Methods). All but 3 of the 33 plant and animal proteins have at least 3 of the 8 motifs outlined in Table 2.

**Fig 2:**
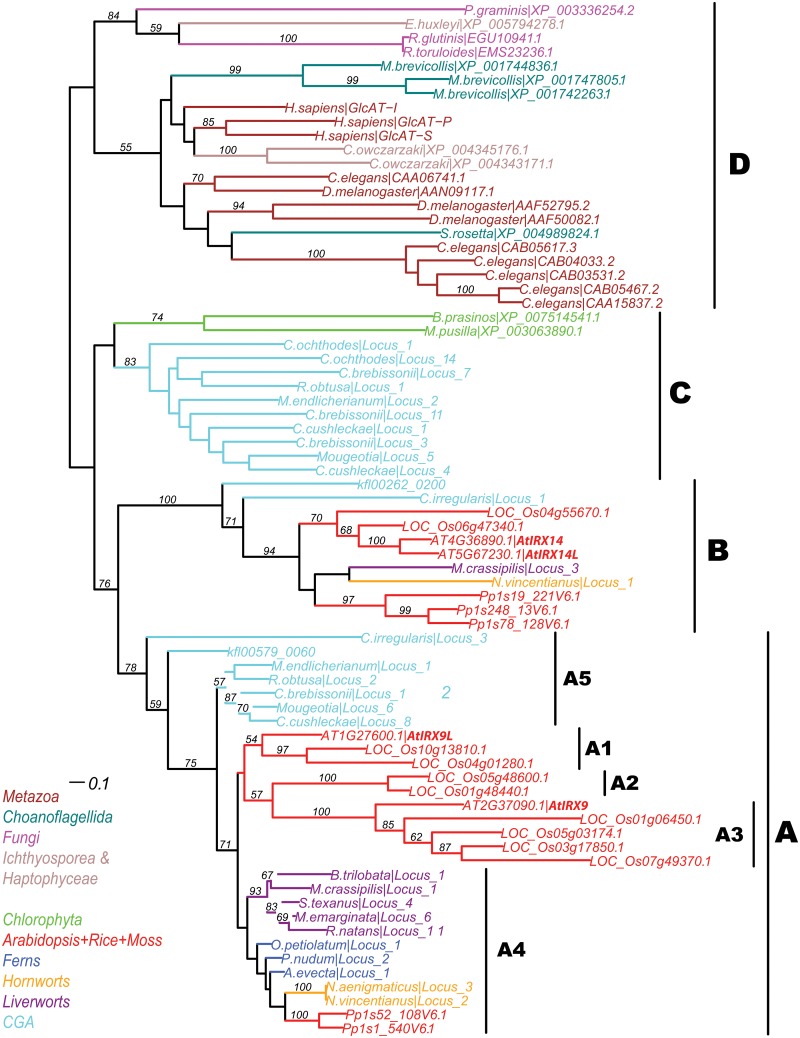
Phylogeny of 74 GT43 proteins. 21 plant proteins are from the PlantCAZyme database, 12 animal proteins are from the CAZy database, 12 early eukaryotic proteins are from Table 1 and 29 early plant transcriptome-derived peptides are from S4 Table. Sequences were aligned using the MAFFT program. Phylogeny was inferred using RAxML program (see Methods). Complete species names are available in S3 Table and S4 Table.

It should be noted that XP_003063890.1 hit the GT43 HMM with an E-value = 2.3e-22 and also CAI63866.1 (a GT43 protein) of the CAZy database as the best hit (6.4e-05) (Table 1). It has less than 3 of the 8 motifs due to its relatively short sequence length (227 aa). We included it in the phylogenetic analysis because it has already been reported [14]. As expected, it was stably clustered with another chlorophyte green algal protein (XP_007514541.1 from *Bathycoccus prasinos*) in the phylogeny (Fig 2). Since both proteins are computationally predicted, future experimental study is needed to confirm whether these chlorophyte proteins are indeed expressed and functional in these chlorophyte algae.

We have verified the reliability of this phylogeny (Fig 2) by including all 54 homologs in (S4 Table) and by editing the sequence alignment to remove regions containing large gaps and ambiguously aligned regions. This effectively eliminated the effect of short fragments on the resulting phylogeny. The new phylogeny (S2 Fig) looks very similar to the one in Fig 2, suggesting that including short fragments did not significantly change the phylogenetic clustering.

### Classification of GT43 proteins of various organisms into four major clades

In order to follow the previous nomenclature used in the plant literature (i.e. A for AtIRX9/9L and B for AtIRX14/14L) and incorporate the newly identified GT43 proteins in various organisms, we suggest a classification of the GT43 family into four clades: A, B, C and D, according to Fig 2. Clade A and B contain land plants and CGA proteins (detailed below), following the two recent reports [12, 13]. These two clades are clustered together with a bootstrap value = 76%, with clade C basal to them. Clade C and D are newly designated clades. Clade C contains exclusively CGA proteins and two *Chlorophyta* proteins, although the grouping of the *Chlorophyta* proteins with CGA proteins is not well supported (bootstrap value < 50%). Clade D only contains non-plant proteins including those from animals, fungi, *Haptophyceae*, *Choanoflagellida*, *Ichthyosporea* and *Haptophyceae*. The function of clade D proteins might be related to the synthesis of proteoglycans, as human GT43 proteins have been known to be involved in the synthesis of a surface carbohydrate epitope HNK1 and glycosaminoglycan using UDP-glucuronic acid as the sugar donor [25]. In contrast proteins of the plant clades A and B are likely to use completely different sugar donors (UDP-xylose) and acceptors (oligosaccharide with β-1,4 xylan backbone).

### GT43 proteins are sporadically distributed in non-plant/animal groups

The early eukaryotic GT43 homologs in clade D hit the GT43 domain model with E-value < 1e-10 and have conserved sequence motifs that are signatures of the GT43 family. Therefore we may conclude that GT43 is more broadly distributed than was once believed. On the other hand, interrogating ~400 completed fungal genomes and a dozen *Chlorophyta* green algal genomes did not reveal more GT43 proteins. Hence the phyletic distribution of GT43 proteins is at most sporadic in early eukaryotes, while they are almost universally present in all sequenced animals and plants (land plants and CGA). Though phylogenetically closer to animal proteins, the origin of these early (non-plant and non-animal) eukaryotic proteins remains a puzzle, potentially as a result of complex evolutionary histories involving horizontal gene transfer and gene loss.

### CGA have three major clades of GT43 proteins and clade C evolved earliest

A recent study has found 16 assembled GT43 EST transcripts in seven out of 13 surveyed CGA transcriptomes [13]. Only two of the 16 transcripts were used in their phylogenetic analysis (others are too short to use). The analysis showed that one was clustered in clade A and the other was clustered in clade B. In our phylogeny (Fig 2), we included 19 CGA sequences, 17 of which are from seven CGA transcriptomes. The other two (kfl00579_0060, kfl00262_0200) are from the recently sequenced *Klebsormidium flaccidum* genome [26]. While we confirmed that CGA proteins fell into both clades A and B, ten of the 19 sequences form the newly defined clade C, ancestral to both A and B. The ten sequences are from six CGA species, suggesting that clade C is broadly distributed in CGA. Although the fully sequenced *K*. *flaccidum* genome contains only A and B proteins, three CGA species including *Cylindrocystis cushleckae*, *Cylindrocystis brebissonii* and *Mougeotia sp*. have representative genes in all the three clades (S2 Fig). Taking transcripts from *C*. *brebissonii* as an example, its clade C transcript C.brebissonii|Locus_3 (translated peptide 254 aa) shares 27% identity with AtIRX9 and 26% identity with AtIRX14; as a comparison, its clade A transcript C.brebissonii|Locus_12 (translated peptide 211 aa) shares 40% identity with AtIRX9, and its clade B transcript C.brebissonii|Locus_4 (translated peptide 185 aa) shares 33% identity with AtIRX14. This supports the phylogeny that clade C is ancestral to both clade A and clade B and represents the most ancient subfamily of plant GT43 genes.

Biochemical evidence has been shown that xylan is present in CGA [27] and it was proposed that CGA have already evolved the enzymatic components for xylan synthesis [13]. Since clade A and B that are involved in xylan synthesis are present in CGA, it remains a highly interesting question what functional role the ancestral clade C might have, especially for species having all three forms of GT43 genes. Future experiments are needed to characterize clade C genes in order to understand their function. The clustering of the two *Chlorophyta* GT43 in clade C suggests they might be also involved in xylan synthesis, given that xylans are also found in *Chlorophyta* [28].

### Angiosperm proteins of clade A could be further classified into three subclades

It is clear from Fig 2 that land plants have two major GT43 clades (A and B), which is consistent with previous reports [12–14]. Clade A contains AtIRX9/AtIRX9L orthologs and clade B contains AtIRX14/AtIRX14L orthologs in all plant taxa spanning from CGA to angiosperms. AtIRX14L is very similar (70% amino acid identity) to AtIRX14; the two proteins must have evolved from a recent duplication event. However AtIRX9 and AtIRX9L are quite different in sequence (39% amino acid identity); therefore in some studies AtIRX9 and AtIRX9L were placed into two different clades [10] or subclades [14].

**Fig 3:**
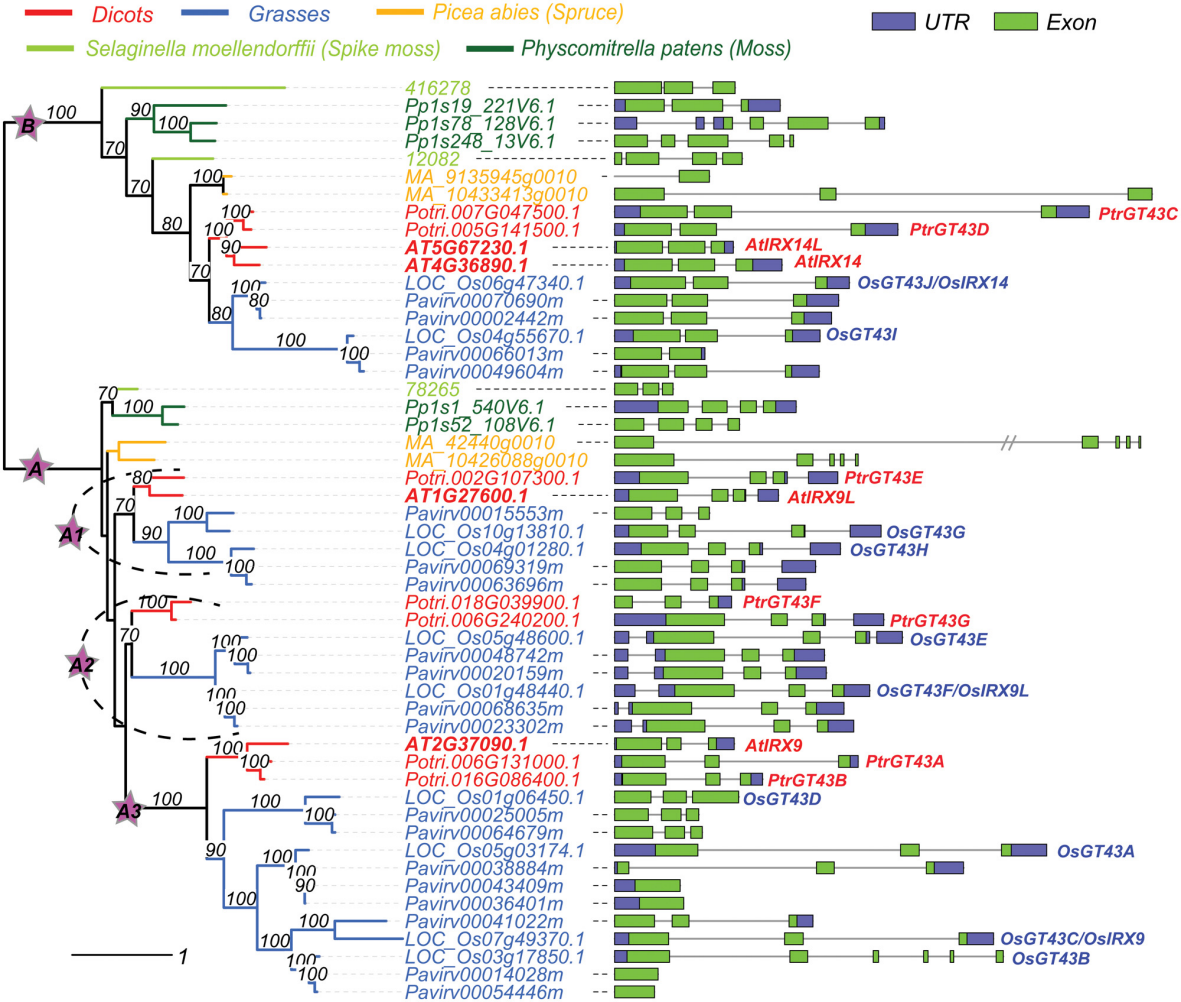
Intron-exon structures of plant GT43 genes. 52 proteins were used to build the phylogeny using RAxML program (see Methods). Gene structures were plotted on the right using the GSDS server [46]. All the protein IDs can be searched at Phytozome [34]. Poplar gene names (e.g. PtrGT43A) were adopted from [47] and rice gene names (e.g. OsGT43A) were adopted from [48].

Similar to many other GT families, GT43 is expanded in plants by gene duplication and sequence divergence. Such expansion is more evident in clade A than B, which makes it necessary to further classify A into subclades. In Fig 2, A is further classified into five subclades. A1, A2 and A3 are angiosperm-specific. A1 contains AtIRX9L, A3 contains AtIRX9 and A2 contains no Arabidopsis proteins. A4 contains proteins from earlier emerging land plants including ferns, hornworts, liverworts and mosses, and A5 is CGA-specific. Although it seems unnecessary to have such a subclade classification given the small number of proteins in each subclade, the clustering appears to be more significant when more land plant proteins are included, as clade A becomes much larger and well separated than B (Fig 3). In addition A2 actually consists of proteins from both grasses and dicots when more species are included in the phylogeny (Fig 3).

### The three land plant subclades have distinct sequence features and expression profiles

According to Figs 2 and 3, the separation of AtIRX14/AtIRX14L (clade B) and AtIRX9L/9 (clade A) predated the emergence of land plants 430~470 million years ago [29], as both clades contain CGA proteins; however, the divergence of AtIRX9L (subclade A1) and AtIRX9 (subclade A3) occurred much later, most likely after the emergence of angiosperms 167~199 million years ago [30], because all three subclades only contain angiosperm proteins.

Among the three subclades, A3 (AtIRX9) has the longest branches (Figs 2 and 3) signifying the most rapid sequence divergence. This is in agreement with findings made in more detailed sequence and expression analyses. For example, AtIRX9 has a smaller number of conserved substrate binding residues than AtIRX9L and AtIRX14 (S2 Table). A3 proteins also have more variable intron-exon gene structures (Fig 3) and more variable sequence lengths (S5 Table). In addition S5 Table shows that grasses have significantly more GT43 genes than other plants especially in subclade A3, consistent with the fact that the cell walls of grasses have higher xylan content [31]. Clade B proteins (AtIRX14/14L) are always the longest and in many cases A3 (AtIRX9) proteins are the shortest. As for GC content, in dicots subclade A3 seems to be the lowest while in grasses subclade A2 is the lowest. These observations tend to be true even when more flowering plants were examined.

**Fig 4:**
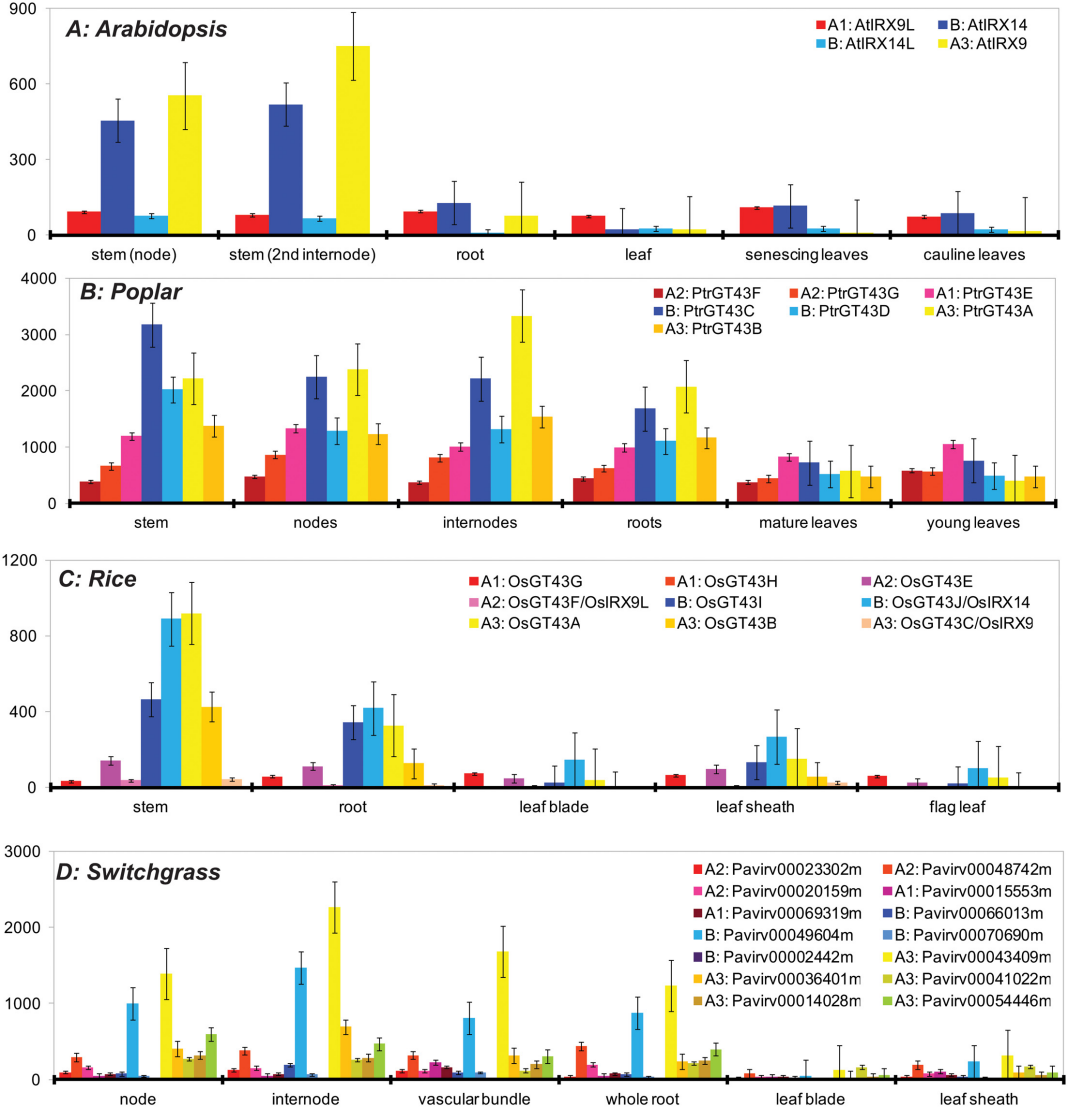
Expression profiles of GT43 genes. In Arabidopsis (A), poplar (B), rice (C) and switchgrass (D). Some genes do not have any probes in the microarray data and were excluded. Inset legends, A1, A2, A3 and B are the clade names followed by the gene names according to Fig 2 and Fig 3. For each tissue, genes are ordered and colored based on their clades: A in red, B in blue and C in yellow. The y-axis shows the expression values from microarray data. A complete version of this figure is available as S3 Fig.

In Arabidopsis and rice, A3 genes (yellow) also have the most extreme change in expression (Fig 4): with highest expression in stem tissues but lowest expression in other tissues. A1 and A2 (red), although phylogenetically close to A3, tend to be expressed at lower levels but almost evenly and widely expressed across all tissues. The more distantly related clade B (blue) has a similar profile as A3 but the differential expression change is less drastic than A3. These observations are also made in poplar [32] and switchgrass (Fig 4 and S3 Fig) with more tissues included. Altogether these data suggest rapid sequence and expression evolution in clade A particularly in subclade A3.

## Materials and Methods

### Sequence and microarray data sources

GT43 protein sequences of fully sequenced land plant genomes were downloaded from PlantCAZyme database [24], which we recently developed. Briefly, protein sequences in PlantCAZyme were collected by running HMMER 3.0 [33] using the GT43 signature domain hidden Markov model (HMM) as the query [18] to search against fully sequenced plant genomes from the Phytozome database [34].

For Arabidopsis, GEO microarray data sets GSE5629-GSE5634 from the GEO database were used (http://arabidopsis.info/). For poplar, GSE6422 was used [35]; for rice, GSE21494 was used [36]; and for switchgrass, microarray data from [37] was used.

### Pipeline for data mining of RNA-Seq data

The computational pipeline shown in S1 Fig was used to retrieve homologous reads to GT43 proteins and to assemble them into longer transcripts for further motif and phylogenetic analyses. Details about the pipeline were explained in [23]. In this new pipeline we used Velvet [38] and Oases [39] to assemble the Illumina reads. VelvetOptimiser (http://www.vicbioinformatics.com/software.velvetoptimiser.shtml) was used to optimize the assembly parameters as suggested by [40]. Only assembled transcripts that led to peptide sequences longer than 100 amino acids and matching GT43 proteins of PlantCAZyme database (E-value < 1e-10) and containing GT43 domains (E-value < 1e-10) were kept for further analyses.

### Phylogenetic analysis

All the multiple sequence alignments (MSAs) were generated using MAFFT v7.158b with the L-INS-i method [41], which is among the most accurate sequence alignment algorithms. Unless specifically indicated (e.g. S2 Fig), all MSAs were not manually edited, because manually editing sequences are rather subjective and impossible for others to reproduce. All phylogenies were built using RAxML 8.0 [42] with the following parameters: 100 times rapid bootstrap analysis and search for best-scoring maximum likelihood tree (-# 100-f a); JTT substitution model, GAMMA model of rate heterogeneity with estimate of proportion of invariable sites (-m PROTGAMMAIJTT). This parameter setting with the maximum likelihood algorithm implemented by RAxML is considered to be one of the most sophisticated and accurate protein phylogeny reconstruction methods.

### Motif analysis

To scan GT43 homologous proteins and peptides, we have derived 8 PROSITE-style motif patterns (Table 1) according to two previous reports [11, 43]. We then used ScanProsite tool [44] to scan the 8 motif patterns in the 21 NCBI-nr protein sequences and 54 peptide sequences from plant transcriptomes. In addition, we also put another constraint on the relative location of the motifs: (i) motifs 1 and 2 must appear in the first half (N-terminal) of the full length sequence; (ii) motifs 7 and 8 must appear in the second half (C-terminal) of the full length sequence and (iii) the rest motifs must appear in the middle part of the sequence.

## Supporting Information

S1 Fig Computational pipeline for transcriptome data mining for GT43 proteins. (EPS)

S2 Fig Phylogeny based on edited sequence alignment of 99 GT43 proteins (21 from plant genomes + 12 from metazoa in CAZy + 12 from NCBI-nr early eukaryotes + 54 early plant transcriptomes) by removing long gaps and ambiguously alignment regions. (EPS)

S3 Fig Expression profiles of GT43 genes in Arabidopsis (A), poplar (B), rice (C) and switchgrass (D). This is a more complete version of Fig 4 with more tissues. (EPS)

S1 Table 21 NCBI-nr proteins that are non-plant and non-animal proteins and hit GT43 domain with E-value < 1e-10; proteins having at least 3 motifs are shown in yellow background. (XLSX)

S2 Table Key residues in human GlcAT-I protein reported in [11, 21] and the corresponding residues in other protein included in Fig 1. (XLSX)

S3 Table 25 Illumina RNA-Seq datasets that are used in data mining of GT43 genes. (XLSX)

S4 Table 54 assembled transcripts that hit their best PlantCAZyme match with E-value < 1e-10; Clade column is based on Fig 2; peptides having at least 3 motifs are shown in yellow background. (XLSX)

S5 Table GT43 genes in seven land plants. (XLSX)
